# Compressional behaviors of ammonium phosphomolybdate hydrate (APMH) with different pressure media

**DOI:** 10.1080/14686996.2025.2580926

**Published:** 2025-10-28

**Authors:** Junhyuck Im, Soojin Lee, Hyunseung Lee, Pyosang Kim, Hyeonsu Kim, Sunki Kwon, Donghoon Seoung, Yongmoon Lee

**Affiliations:** a Decommissioning Technology Research Division, Korea Atomic Energy Research Institute (KAERI), Daejeon, Korea; b Department of Geological Sciences, Pusan National University, Busan, Korea; c Department of Earth and Environmental Sciences, Chonnam National University, Gwangju, Korea; d Institute for Future Earth Environment, Pusan National University, Busan, Republic of Korea

**Keywords:** High-pressure, compressibility, bulk moduli, ammonium phosphomolybdate hydrate, synchrotron X-ray powder diffraction, pressure-transmitting media

## Abstract

This study investigates the pressure-dependent structural response of ammonium phosphomolybdate hydrate (APMH) under four distinct pressure-transmitting media (PTMs): distilled water, methanol, ethanol, and silicone oil. Synchrotron X-ray diffraction combined with Rietveld refinement confirmed that APMH maintains the archetypal Keggin-type framework while incorporating approximately ten crystallographic water molecules per unit cell, distributed over two distinct coordination sites (OW1 and OW2). High-pressure diffraction experiments revealed pronounced PTM-dependent compressional behaviors. In water, APMH undergoes an abrupt 2.6% volume collapse near 2 GPa followed by framework stiffening, while silicone oil induces significant densification above ~4 GPa. By contrast, methanol and ethanol promote smooth, elastic contraction without discontinuities. Bulk moduli derived from equation-of-state fitting span a wide range, from ~28 GPa under low-pressure silicone oil to 321 GPa at high pressures, highlighting the critical role of PTM chemistry and penetrability. Microstrain analysis further identified anisotropic deformation, with the (222) planes particularly sensitive to stress accumulation under both water and silicone oil. These results demonstrate that APMH compressibility is not an intrinsic constant, but a variable property governed by external medium.

## IMPACT STATEMENT

This study reveals pressure-dependent, medium-specific compressibility of hydrated POMs, highlighting PTM chemistry as key determinants for framework resilience and pressure-responsive material design.

## GRAPHICAL ABSTRACT

**Figure:**
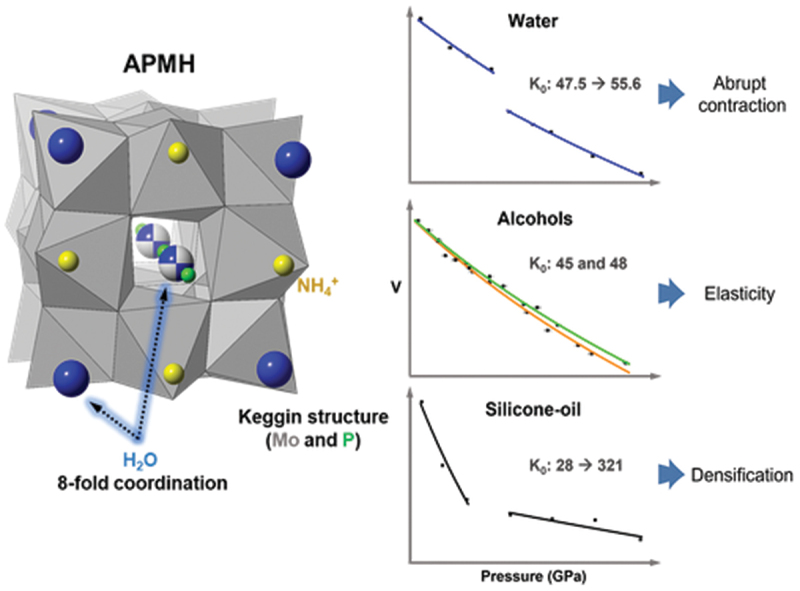

## 1. Introduction

**Figure 1.**
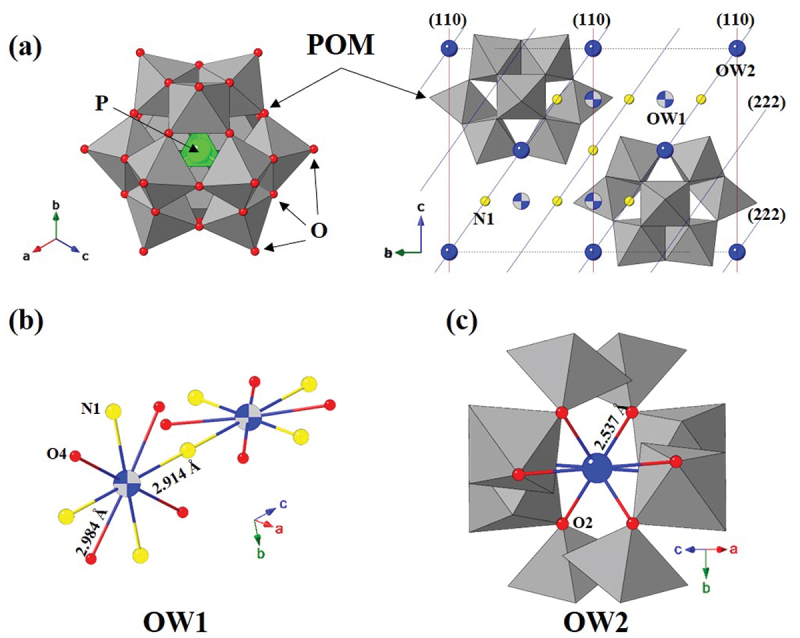
Structural models of APMH: (a) Keggin framework with MoO₆ octahedra in grey, O atoms in red, P atoms in green, and ammonium N in yellow; two water molecule O sites are shown as a blue sphere and a blue beach-ball symbol. The (110) and (222) planes are also shown as the red and blue lines, respectively. (b) Sixfold coordination of OW1 with ammonium N and framework O4. (c) Eightfold coordination of OW2 with framework O2.

Polyoxometalates (POMs) are a diverse class of discrete metal – oxygen clusters composed of early transition metals (Mo, W, V) in their highest oxidation states, connected via shared oxygen atoms to form architecturally well-defined anionic frameworks. Owing to their redox activity, acidity, and structural tunability, POMs have attracted considerable attention for applications in catalysis, ion exchange, photochemistry, and electrochemical energy storage [1–3]. Among the archetypal POM structures, Keggin-type anions such as [PMo₁₂O₄₀]^3−^ remain among the most studied due to their well-preserved symmetry and robust chemical framework [1,4,5]. Ammonium phosphomolybdate (APM), composed of Keggin anions charge-balanced by NH₄^+^ cations, is often used as a benchmark material in POM studies [6–9]. This inorganic exchanger is characterized by high selectivity of cesium sorption even in acid solutions, it possesses sufficient capacity, quick kinetics of ion exchange and high radiation stability [10]. While the crystal structure of anhydrous APM has been well characterized, few are known about its hydrated analogue, ammonium phosphomolybdate hydrate (APMH). In the case of APMH, the presence of water molecules occupying interstitial or coordinated positions within the framework can introduce additional degrees of freedom for pressure-induced structural reorganization (Figure 1). To date, high-pressure studies on APMH and other POMs remain extremely limited [11–13]. Although POMs are well known for their structural versatility and functional tunability, relatively little attention has been devoted to their compressional behavior and pressure-induced phase transitions compared to other porous frameworks, including zeolites, MOFs, and ZIFs. Understanding this response is essential for evaluating their mechanical resilience and potential use in stimuli-responsive applications. High-pressure crystallographic studies on porous and molecular frameworks increasingly emphasize the non-negligible role of the pressure-transmitting medium (PTM) [14–16]. The hydrostaticity, polarity, viscosity, and molecular size of the PTM can drastically affect not only the uniformity of pressure distribution but also the degree to which the medium penetrates and interacts with the host structure. Penetrating PTMs such as water, methanol, and ethanol are known to induce framework swelling, pore filling, or hydrogen-bond mediated reconfigurations. In contrast, bulky, non-polar, and chemically inert media like silicone oil typically preserve framework integrity by applying pore collapse and amorphization, non-penetrating pressure [17–19]. Consequently, the mechanical response of hydrated POMs under pressure cannot be viewed as an intrinsic material property alone, but rather as a composite response involving both internal hydration states and external environmental factors.

In this study, we investigate the pressure-dependent compressional behavior of APMH using synchrotron-based X-ray powder diffraction under four different PTMs: distilled water, methanol, ethanol, and silicone oil. Structural refinement via Rietveld analysis establishes a hydrated Keggin-type model incorporating approximately ten crystallographic water molecules, each occupying distinct coordination environments. Through equation-of-state (EOS) fitting, we identify PTM-dependent compressional regimes, including abrupt volume collapse and mechanical stiffening, particularly under water and silicone oil. By elucidating how hydration and the chemistry of the pressure-transmitting medium influence the mechanical response of APMH, this study advances the understanding of pressure-induced compressibility mechanisms in soft molecular frameworks.

## 2. Materials and methods

### 2.1. Sample

Ammonium phosphomolybdate hydrate (APMH, (NH_4_)_3_[PMo_12_O_40_]·xH_2_O, SIGMA-ALDRICH 342, 165, USA) was ground for powder diffraction experiments. To quantify the number of H₂O molecules in the channels, thermogravimetric analysis (TGA, model SDT500, USA) was performed at the Yonsei Center for Research Facilities in Korea. The measurements were carried out from 25 to 700°C at a heating rate of 10°C min^− 1^ under a nitrogen atmosphere. Graphical result is shown in Figure S2.

### 2.2. High-pressure synchrotron X-ray powder diffraction

*In-situ* high-pressure synchrotron X-ray diffraction experiments were performed at the beamline 10–2 at Stanford Synchrotron Radiation Lightsource (SSRL) and beamline 5A at Pohang Accelerator Laboratory (PAL). At beamline 10–2, a focused X-ray beam with a wavelength of 0.61992(1) Å was used. Collected diffraction patterns of APMH in the presence of silicone-oil PTM were obtained using a Pilatus 300K-w direct photon-counting detector. At beamline 5A at PAL, hard X-ray photons emitted from an in-vacuum undulator source were monochromatized to a wavelength of 0.69265(1) Å, and the APMH in water, methanol and ethanol PTM were detected using MAR345 imaging plate detector. The wavelength of the incident beam and detector calibrations were carried out using LaB_6_ standard reference material (SRM660c) at both synchrotron facilities.

### 2.3. Diamond-anvil cell preparation

A modified 4-pin type diamond anvil cell (DAC) was used for the high-pressure X-ray powder diffraction experiments, equipped with two diamond anvils (0.5 mm culet diameter) and tungsten carbide supports [20]. A stainless-steel gasket of 0.2 mm thickness was pre-indented to a thickness of ca. 0.1 mm and a 0.2 mm sample chamber hole diameter was drilled by an electro-discharge machine (EDM). The powder sample of APMH was placed in the gasket sample hole with ruby chips for *in-situ* pressure calibrations.

Ambient pressure data of APMH were collected on the dry powder sample for non-swelling conditions and the wet powder samples saturated by water molecules for the swelling environment, respectively. Subsequently, the silicone oil and distilled water were added to the sample chamber as a pressure-transmitting media, and the DAC was sealed to the first pressure point. The sample pressure is measured by detecting the shift in the R1 emission line of included ruby chips [21]. The samples were typically equilibrated for about 10 min. in the DAC at each measured pressure point. The pressure was increased in steps of 0.3 – 0.6 GPa up to about 10 GPa in silicone PTM and to about 5–6 GPa in water, methanol and ethanol PTM.

### 2.4. Ambient Rietveld refinement

The ambient structural model was established by Rietveld refinement using the EXPGUI program suite [22–24]. Prior to the refinement, whole-profile fitting was performed by the Le Bail method to extract reliable peak intensities. The background was modeled with a Chebyshev polynomial below 20 coefficients, and the observed Bragg reflections were fitted with the pseudo-Voigt function proposed by Thompson et al. [25]. To reduce the number of refined parameters, isotropic displacement factors were constrained by grouping the framework atoms (Mo, P, and O) separately from the extra-framework species (ammonium N and water O atoms). Geometrical soft restraints were applied to the T – O (*T* = Mo, P) and O – O distances within the polyhedra: P–O bonds were restrained to 1.542 ± 0.001 Å, and Mo–O bonds were restrained to 1.680, 1.914, 1.923, and 2.433 ± 0.001 Å. The O–O distances were restrained to 2.519, 2.606, 2.616, 2.645, 2.682, 2.782, 2.811, and 2.922 ± 0.001 Å, following the parameters reported by Boeyens et al. [26]. In the final refinement stage, these restraints did not lead to any significant changes in the interatomic distances, and convergence was achieved by simultaneously refining all background and profile parameters, scale factors, lattice constants, 2θ zero shift, and the atomic positional and thermal displacement parameters. The final refined parameters are summarized in Tables 1 and 3, and the selected bond distances are listed in Table 2.

**Table 1.** Refined unit-cell parameters and fractional coordinates of APMH under ambient pressure^a,b^.

Pressure (GPa)		0.00, capillary			
Space group		**P***n-3 m*			
Unit cell parameter		*a* = 11.6560(1) (Å), vol = 1583.61(2) (Å^3^)			
Unit cell composition		(NH_4_)_6_(PO_4_)_2_[Mo_12_O_36_]_2_ ·10 H_2_O			
wRp (%)		4.12			
P1 2a	*x*	0.25	N1 12f	*x*	0.75
	*y*	0.25		*y*	0.25
	*z*	0.25		*z*	0
	*U*_*iso*_^*c*^	0.0293(5)		*Occu.*	0.5
Mo1 24k	*x*	0.2605(2)		*U*_*iso*_	0.74(8)
	*y*	0.4671(1)	OW1 6d	*x*	0.75
	*z*	0.4671(1)		*y*	0.75
O1 8 g	*x*	0.3244(5)		*z*	0.25
	*y*	0.3244(5)		*U*_*iso*_	0.045(7)
	*z*	0.3244(5)	OW2 4c	*x*	0
O2 24k	*x*	0.1534(6)		*y*	0.5
	*y*	0.3466(6)		*z*	0.5
	*z*	0.5167(9)			
O3 24k	*x*	0.3723(6)			
	*y*	0.3723(6)			
	*z*	0.5411(9)			
O4 24k	*x*	0.241(1)			
	*y*	0.5691(5)			
	*z*	0.5691(5)			

^a^Estimated standard deviations are given in parentheses.

^b^The occupancies of all atoms are unity, except for N1.

^c^Isotropic displacement factors (*U*_*iso*_) were refined by grouping.

**Table 2.** Selected atomic distances of APMH^a^.

Framework atom sites	P1-O1	1.50(1)
	Mo1-O1	2.467(6)
	Mo1-O2	1.966(3)
	Mo1-O3	1.914(5)
	Mo1-O4	1.697(8)
	O1-O1	2.45(2)
	O1-O2	3.010(9)
	O1-O3	2.65(1)
	O2-O2	2.81(2)
	O2-O3	2.584(7)
	O2-O4	2.85(1)
	O3-O3	2.78(2)
	O3-O4	2.78(1)
Extra-framework atom sites	OW1-O4	2.984(9)
	OW1-N1	2.914(1)
	OW2-O2	2.537(9)

^a^Estimated standard deviations are given in parentheses.

### 2.5. High-pressure equation of state analysis

The pressure-dependent changes in the unit-cell lengths and volumes were subsequently evaluated. The bulk moduli, derived from normalized volume (V/V₀), were calculated using the third-order Birch–Murnaghan equation of state (EOS), Equation (1) [27]. For the bulk modulus calculations, the value of B′ was fixed at 4, as is commonly assumed for natural materials.

(1) P=32B0V0V−7/3−V0V−5/31+34B 0′−4V0V−2/3−1

## 3. Results and discussions

To accurately evaluate the crystallographic structure of APMH at ambient conditions, synchrotron X-ray powder diffraction data were analyzed by Rietveld refinement. The refined unit cell corresponds to a composition of (NH_4_)_6_[PMo_12_O_40_]·10 H₂O, consistent with the hydration level determined by thermogravimetric analysis. Figure 1 shows the refined structural model of APMH, which retains the Keggin-type polyoxometalate framework [PMo₁₂O₄₀]^3−^ with ammonium cations occupying interstitial sites. Within this hydrated framework, two distinct crystallographic water sites were identified. OW1 is 8-fold coordinated with ammonium nitrogen (N1) and framework oxygen (O4), with interatomic distances of 2.914(1) Å (OW1–N1) and 2.984(9) Å (OW1–O4). In contrast, OW2, also 8-fold coordinated, interacts primarily with framework oxygen (O2) with an interatomic distance of 2.537(9) Å (OW2–O2). These distances confirm that water molecules are not randomly distributed but occupy well-defined coordination environments, contributing to the stabilization and subtle expansion of the Keggin framework. The incorporation of ~10 H₂O molecules per unit cell leads to a moderate increase in unit-cell volume (1583.61(2) Å^3^ in this work) compared to the anhydrous analogue (ca. 1556.9 and ca. 1536.8 Å^3^), while preserving the overall topology [7]. This refined structural model provides a robust foundation for analyzing the pressure-dependent compressional behavior of APMH.

**Figure 2.**
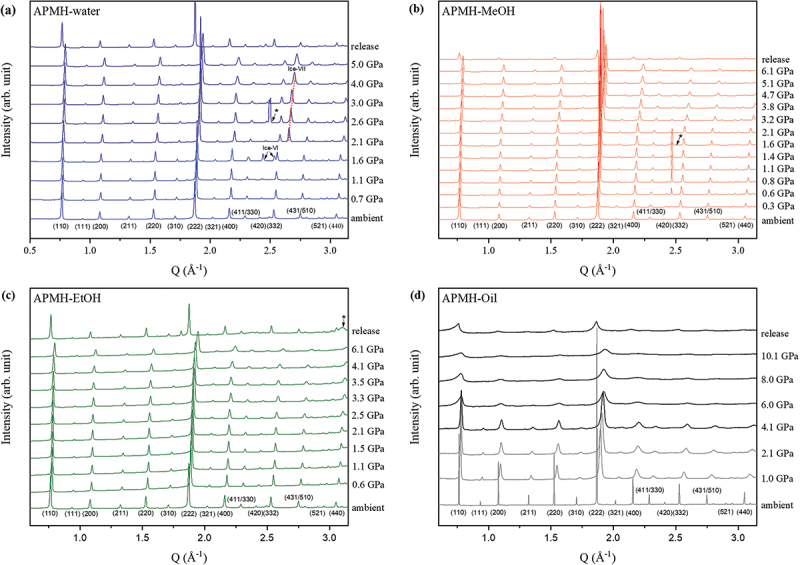
In-situ synchrotron X-ray diffraction patterns of APMH under compression using four pressure-transmitting media: (a) distilled water, (b) methanol, (c) ethanol, and (d) silicone oil. Peak shifts toward higher angles indicate lattice contraction; differences in peak broadening and intensity reflect PTM-dependent structural responses.

When pressure was applied, APMH exhibited strongly PTM-dependent responses (Figure 2). Diffraction peaks shifted to higher angles in all cases, reflecting lattice contraction, but the degree of peak broadening and shift varied significantly with the PTM. In water, pronounced broadening and shift were observed above 2 GPa, particularly at low-angle reflections such as (001). This broadening and shift can be attributed both to framework distortions arising from water penetration and reorganization of hydration layers, and to the phase transformation of the pressure-transmitting medium, as liquid water undergoes solidification under pressure. In methanol and ethanol, peak shifts were more gradual, and reflections remained relatively sharp up to 6 GPa, although ethanol showed slightly stronger attenuation than methanol, consistent with steric hindrance due to its larger molecular size. In silicone oil, unlike in the other runs, the diffraction peaks begin to broaden from the very first pressure step, and above ~6 GPa they become distinctly asymmetric. This behavior suggests that, under a non-penetrating PTM, the framework may undergo abrupt collapse or experience significant microstrain. This behavior is consistent with high-pressure studies on zeolite, MOF, and ZIF systems, which have demonstrated that non-penetrating PTMs often destabilize frameworks through disordering mechanisms such as microstrain and amorphization while penetrating fluids tend to preserve long-range ordering through insertion of guest molecules into framework [28–35].

**Figure 3.**
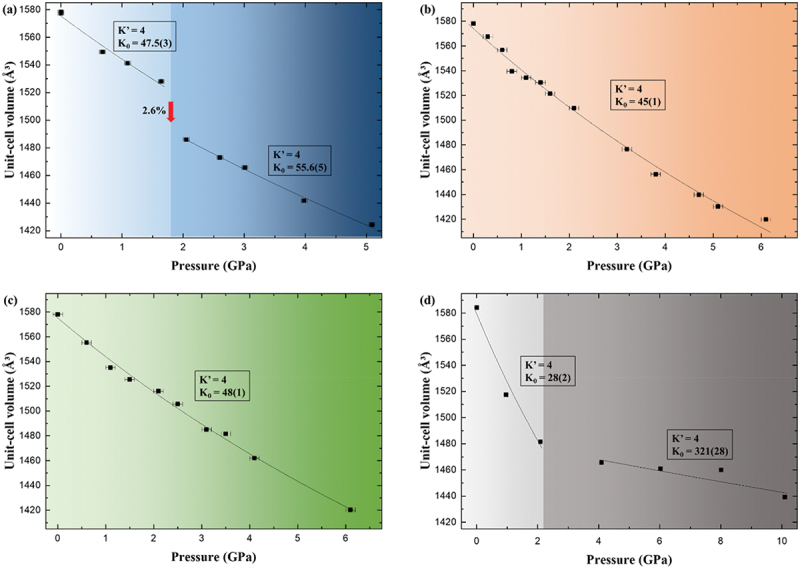
Pressure-dependent unit-cell volume changes and bulk-moduli of APMH in distilled (a) water, (b) methanol, (c) ethanol and (d) silicone PTMs at room temperature.

The pressure – volume relations shown in Figure 3 and summarized in Table 3 further highlight these PTM-dependent differences. In water, the unit-cell volume decreased from 1577.5 Å^3^ at ambient conditions to 1424.5 Å^3^ at 5.1 GPa, corresponding to a 9.7% reduction. At 1.6 GPa, the normalized volume was 0.969, but a sudden 2.6% contraction to 0.943 occurred between 1.6 and 2.1 GPa. After this abrupt transition, the framework stiffened, with the bulk modulus increasing from 47.5(3) GPa at lower pressures to 55.6(5) GPa beyond 2.1 GPa. This stepwise collapse resembles pressure-induced hydration and non-framework cation migration observed in natrolite-type zeolites [28,29]. By contrast, in methanol and ethanol, compression progressed smoothly and monotonically, with normalized volumes of 0.957 and 0.961 at 2.1 GPa and bulk moduli of 45–48 GPa. Such elastic, reversible behavior is comparable to the breathing-type deformation reported in flexible MOFs under pressure [30,31]. In silicone oil, APMH initially compressed softly up to 2.1 GPa with a normalized volume of 0.935 and a bulk modulus of 28(2) GPa, but beyond 4 GPa the bulk modulus increased abruptly to 321(28) GPa, reflecting a pore-collapse densification phenomenon similar to that documented for ZIFs and siliceous zeolites under inert PTMs [17,32].

**Table 3.** Refined unit-cell parameters under different pressure-transmitting media^a^.

Pressure (GPa)	Water	
	axis length (Å)	Unit cell Volume (Å^3^)
0	11.6409 (2)	1577.5 (1)
0.7 (1)	11.5715 (2)	1549.4 (1)
1.1 (1)	11.5513 (2)	1541.3 (1)
1.6 (1)	11.5181 (3)	1528.1 (1)
2.1 (1)	11.4115 (2)	1486.0 (1)
2.6 (1)	11.3781 (3)	1473.0 (1)
3.0 (1)	11.3593 (4)	1465.7 (1)
4.0 (1)	11.2970 (4)	1441.8 (1)
5.1 (1)	11.2517 (4)	1424.5 (2)
0^b^	11.6432 (5)	1578.4 (2)
Pressure (GPa)	Methanol	
	axis length (Å)	Unit cell Volume (Å^3^)
0	11.6428 (3)	1578.2 (2)
0.3 (1)	11.6163 (3)	1567.5 (1)
0.6 (1)	11.5899 (4)	1556.8 (2)
0.8 (1)	11.5467 (3)	1539.5 (1)
1.1 (1)	11.5337 (4)	1534.3 (1)
1.4 (1)	11.5241 (4)	1530.5 (2)
1.6 (1)	11.5017 (4)	1521.5 (2)
2.1 (1)	11.4720 (4)	1509.8 (2)
3.2 (1)	11.3869 (4)	1476.5 (2)
3.8 (1)	11.3349 (4)	1456.3 (2)
4.7 (1)	11.2917 (3)	1439.7 (1)
5.1 (1)	11.2668 (4)	1430.2 (2)
6.1 (1)	11.2395 (4)	1419.8 (1)
0^b^	11.6474 (4)	1580.1 (2)
Pressure (GPa)	Ethanol	
	axis length (Å)	Unit cell Volume (Å^3^)
0	11.6428 (3)	1578.2 (2)
0.6 (1)	11.5862 (3)	1555.3 (1)
1.1 (1)	11.5357 (4)	1535.1 (2)
1.5 (1)	11.5113 (5)	1525.6 (2)
2.1 (1)	11.4880 (4)	1516.1 (2)
2.5 (1)	11.4614 (4)	1505.6 (2)
3.1 (1)	11.4090 (4)	1485.1 (2)
3.5 (1)	11.4001 (5)	1481.6 (2)
4.1 (1)	11.3497 (5)	1462.0 (2)
6.1 (1)	11.2406 (9)	1420.3 (3)
0^b^	11.6441 (6)	1578.8 (2)
Pressure (GPa)	Silicone oil	
	axis length (Å)	Unit cell Volume (Å^3^)
0	11.6576 (2)	1584.3 (1)
1.0 (1)	11.4696 (2)	1508.8 (1)
2.1 (1)	11.4001 (2)	1481.6 (1)
4.1 (1)	11.3592 (6)	1465.7 (2)
6.0 (1)	11.347 (1)	1461.0 (4)
8.0 (1)	11.345 (1)	1460.0 (5)
10.0 (1)	11.290 (2)	1439.2 (7)
0^b^	11.727 (3)	1612 (1)

^a^Estimated standard deviations are given in parentheses.

^b^Phases recovered at ambient pressure.

**Figure 4.**
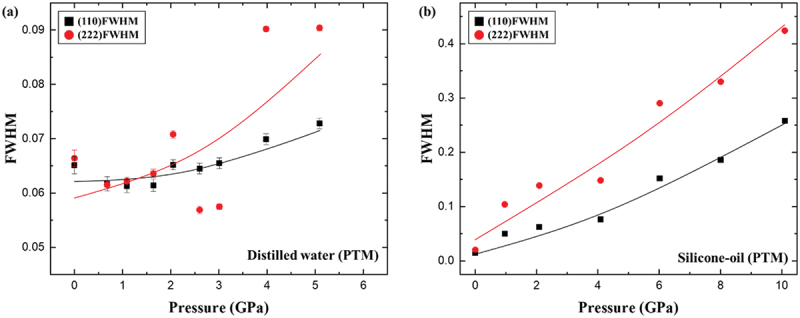
Pressure-dependent FWHM changes of the interplane (110) and (222) bragg reflections of APMH under (a) distilled water and (b) silicone oil environments, respectively.

Microstrain diagnostics from FWHM analysis (Figure 4) provide further insight into anisotropic deformation. In water, both the (110) and (222) reflections broadened gradually with pressure, but above 2 GPa the (222) reflection broadened more rapidly, reaching 0.0901 at 5.1 GPa compared to 0.0684 for (110). This divergence coincided with the abrupt volume collapse and suggested that stress accumulation was concentrated along crystallographic directions intersecting the hydration layers. In silicone oil, reflections began much sharper, with initial FWHM values of 0.0147 and 0.0203 for (110) and (222), respectively, but the (222) reflection broadened steeply to 0.158 at 6 GPa, while the (110) remained near 0.07. This anisotropy indicates that (222) planes are more vulnerable to local strain, paralleling reports of directional collapse in zeolites and ZIF-8, where solvent-accessible crystallographic planes deform preferentially under compression [32,36].

The collective results demonstrate that APMH undergoes PTM-dependent, multi-regime compression. In water, the framework experiences abrupt volume contraction and reorganization driven by strong fluid – framework interaction. In alcohols, the structure contracts smoothly and elastically without significant disruption. In silicone oil, the framework remains crystalline at low pressures but stiffens markedly at higher loads, reflecting pore densification in the absence of fluid penetration. Across all PTMs, the bulk modulus values span from 28 to 321 GPa, much broader than the typical ranges for zeolites (ca. 25–50 GPa), MOFs (ca. 5–20 GPa), and ZIFs (ca. 1–17 GPa) [17,30,31,33,37–39].

Remarkably, APMH fully recovers its initial unit-cell volume after decompression under all PTMs. Even in silicone oil, where partial amorphization and framework collapse occur above 6.0 GPa, the structure nonetheless returns to its original ambient volume once the load is released. This complete recovery, despite transient disorder, contrasts sharply with many MOFs and ZIFs that undergo irreversible amorphization and fail to regain their initial structural state under comparable conditions [30,32,34–36,40].

## 4. Conclusion

Synchrotron X-ray diffraction combined with Rietveld refinement revealed that ammonium phosphomolybdate hydrate (APMH) retains the archetypal Keggin-type framework while incorporating two distinct hydration sites, OW1 and OW2, within its lattice. High-pressure experiments demonstrated that the compressional response of APMH is strongly dependent on the pressure-transmitting medium (PTM): distilled water induces a two-step compression with an abrupt volume collapse near ~2 GPa followed by stiffening, methanol and ethanol promote smooth elastic contraction without discontinuities, and silicone oil preserves structural order at low pressures but triggers significant densification beyond ~4 GPa. Microstrain analysis further showed anisotropic deformation localized along the (222) crystallographic planes under both water and silicone oil. Importantly, the present findings underscore that the compressibility of hydrated POMs is not an intrinsic property but is governed by external PTM chemistry. More broadly, APMH emerges as a unique model system capable of reproducing pressure-response modes characteristic of distinct material classes: zeolite-like hydration-driven collapse, MOF/ZIF-like elastic contraction or densification. This highlights its potential as a platform for designing pressure-responsive functional materials. Furthermore, the insights gained here provide valuable guidelines for high-pressure crystallography, emphasizing the crucial role of PTM selection in experimental design, and suggest broader applications of APMH, and even POM analogues, in areas where controllable compressibility and recovery are essential, such as mechanical damping, energy storage, and adaptive catalysis. Future work will include detailed structural modeling and DFT-based energy analyses to further clarify enthalpy changes and transition mechanisms.

## Supplementary Material

Supplemental Material
