# Supplementary material for: Compressional behaviors of ammonium phosphomolybdate hydrate (APMH) with different pressure media
Source: Sci Technol Adv Mater. 2025 Oct 28;26(1):2580926. doi: 10.1080/14686996.2025.2580926 (PMC12667314; doi:10.1080/14686996.2025.2580926)

**Supporting Information**

**Compressional behaviors of ammonium phosphomolybdate hydrate (APMH) with different pressure media**

Junhyuck Im^1^, Soojin Lee^2^, Hyunseung Lee^2^, Pyosang Kim^3^, Hyeonsu Kim^3^, Sunki Kwon^4^, Donghoon Seoung^3,*^, and Yongmoon Lee^2,4,*^

*^1^Decommissioning Technology Research Division, Korea Atomic Energy Research Institute (KAERI), Daejeon 34057, Korea; ^2^Department of Geological Sciences, Pusan National University, Busan 46241, Korea; ^3^Department of Earth and Environmental Sciences, Chonnam National University, Gwangju 61186, Korea; ^4^Institute for Future Earth Environment, Pusan National University, Busan 46241, Republic of Korea*

*Corresponding authors:

Yongmoon Lee

E-mail: [lym1229@pusan.ac.kr](mailto:lym1229@pusan.ac.kr)

Tel: +82-51-510-2254

Fax: +82-51-517-6389

Donghoon Seoung

E-mail: [dseoung@jnu.ac.kr](mailto:dseoung@jnu.ac.kr)

Tel: +82-62-530-3452

Fax: +82-62-530-3459

**Figure S1.** Graphical result of APMH using Rietveld refinement


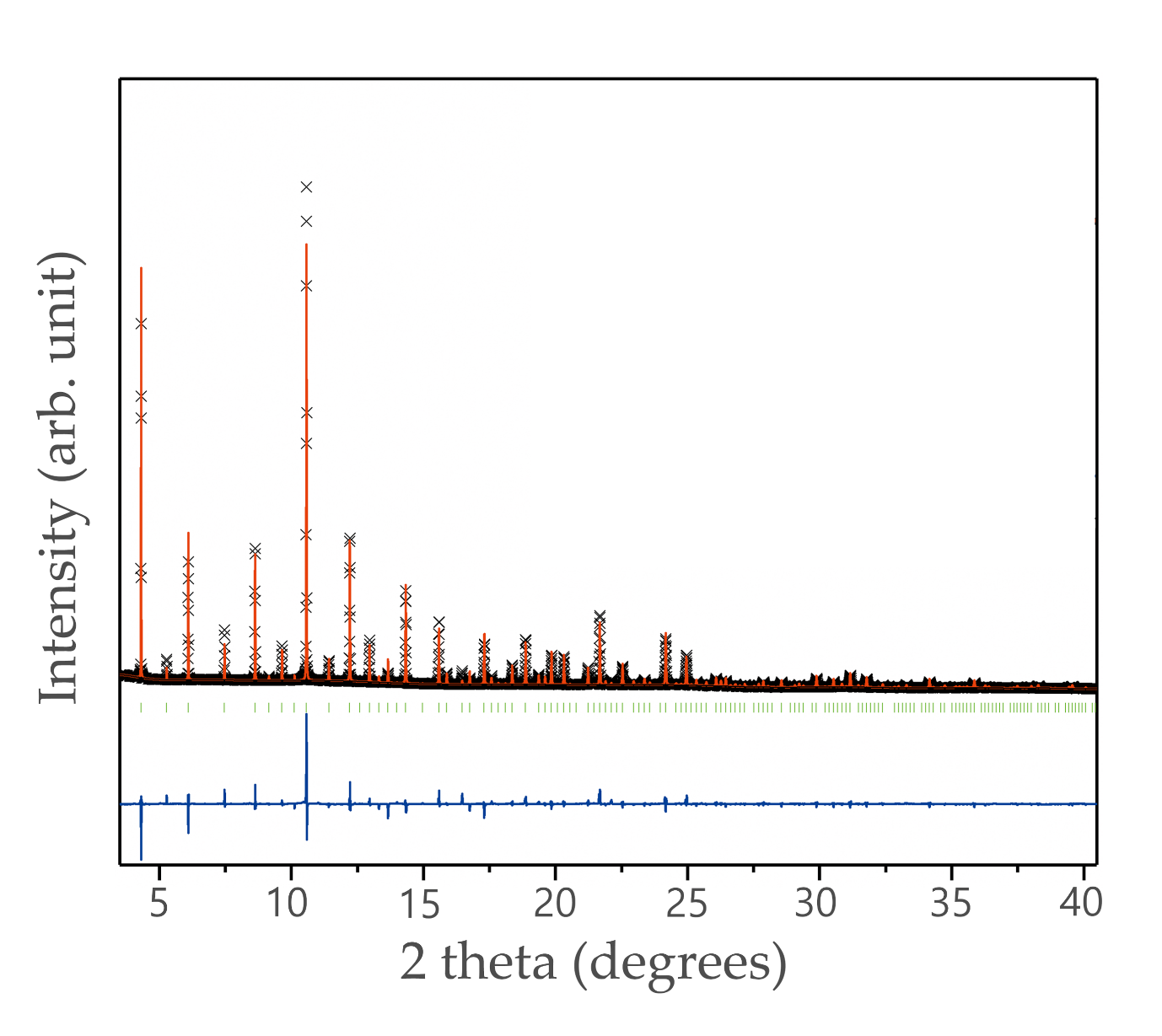


**Figure S2.** Thermogravimetric result of as-prepared APMH


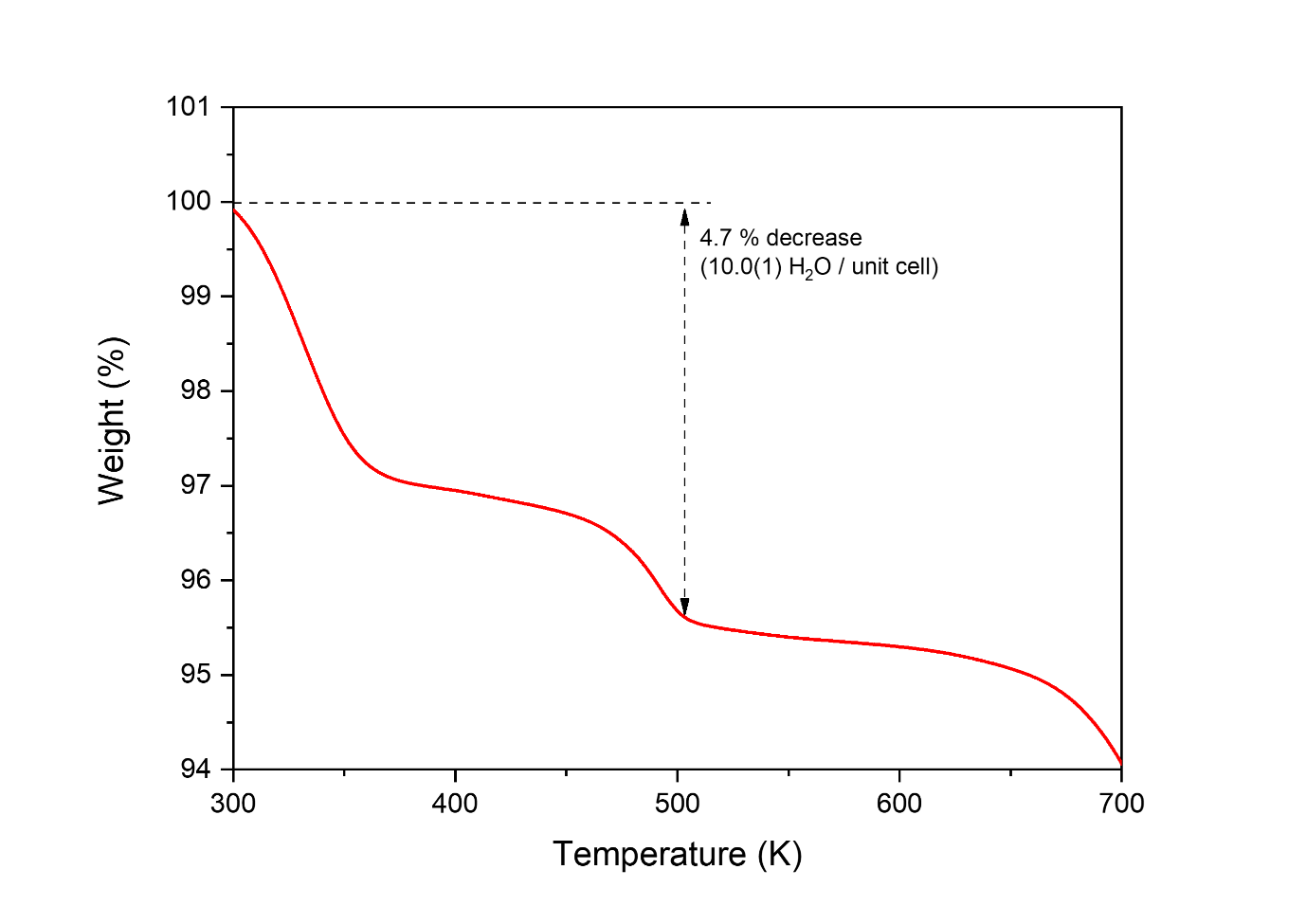

Supplement: Supplemental Material [file TSTA_A_2580926_SM3987.docx]
